# Genome sequence of lytic phage phi1_164023 targeting ST23 KL1-type carbapenem-resistant *Klebsiella pneumoniae*

**DOI:** 10.1128/mra.00139-26

**Published:** 2026-04-20

**Authors:** Huan Kuang, Qingqing Fang, Yu Feng, Zhiyong Zong

**Affiliations:** 1 Center of Infectious Diseases, West China Hospital, Sichuan University 12530, Chengdu, China; 2 Department of General Practice, General Practice Medical Center, West China Hospital, Sichuan University 12530, Chengdu, China; 3 Laboratory of Pathogen Research, West China Hospital, Sichuan University 12530, Chengdu, China; 4 Division of Infectious Diseases, State Key Laboratory of Biotherapy, Chengdu, China; 5 State Key Laboratory of Respiratory Health and Multimorbidity, Chengdu, China; University of Maryland Baltimore, Baltimore, Maryland, USA

**Keywords:** *Klebsiella pneumoniae*, hypervirulent, carbapenem resistance, phage, phage therapy

## Abstract

We report the genome of a phage that belongs to the *Przondovirus* genus, which was isolated from sewage, and is capable of lysing ST23 KL1-type carbapenem-resistant *Klebsiella pneumoniae* strains. The genome is 39,059 bp in length, with a GC content of 53.11%, consisting of 55 protein-coding sequences and no tRNAs.

## ANNOUNCEMENT

The World Health Organization warns of global spread of hypervirulent *Klebsiella pneumoniae* sequence type 23 (ST23) and capsular type KL1 strain, with cases in at least 12 countries (1). These strains have acquired resistance to last-line antimicrobial agents such as carbapenems, thereby positioning bacteriophage (phage) therapy as a promising alternative. Here, we report the genome of phage phi1_164023, a member of the *Przondovirus* genus, which demonstrates effective lytic activity against clinical isolates of ST23 KL1 carbapenem-resistant *K. pneumoniae* (CRKP) with the potential to be hypervirulent.

In January 2024, phi1_164023 was isolated from untreated sewage at the wastewater treatment station of a local hospital. Phages were enriched from sewage using the ST23 KL1 hvCRKP host (37°C, 4 h) and purified by triple double-layer agar plating, as previously described (2). The study was approved by the Ethical Committee of West China Hospital. The genomic DNA of phi1_164023 was extracted from purified phage particles using a phage DNA isolation kit (Norgen Biotek, Sorod, Canada) according to the manufacturer’s instructions. The sequencing library was prepared using the NEBNext Ultra II DNA Library Preparation Kit (New England Biolab, Ipswich, MA). The DNA was first sheared to 350 bp with a Covaris LE220R-plus ultrasonicator, followed by purification and size selection using Agencourt SPRIselect beads. Sequencing was performed on an Illumina NovaSeq 6000 platform. The original sequencing reads were subjected to quality control by Trimmomatic v0.39 (3), with adapter sequences removed and reads below 130 bases discarded. Genome assembly was completed using Unicycler v0.5.0 (4). CheckV 1.0.3 (5) was employed to assess contamination and exclude non-phage conjugates. Genomic annotation was performed using Pharokka v1.7.2 (6), and BLAST was used to identify the phage with the highest overall DNA similarity (identity × coverage) (7). Antimicrobial resistance and virulence genes were identified using the CARD v4.0.1 (8) and VFDB (http://www.mgc.ac.cn/VFs/) (9) databases, respectively. Unless otherwise specified, the software runs with default parameters.

**Fig 1:**
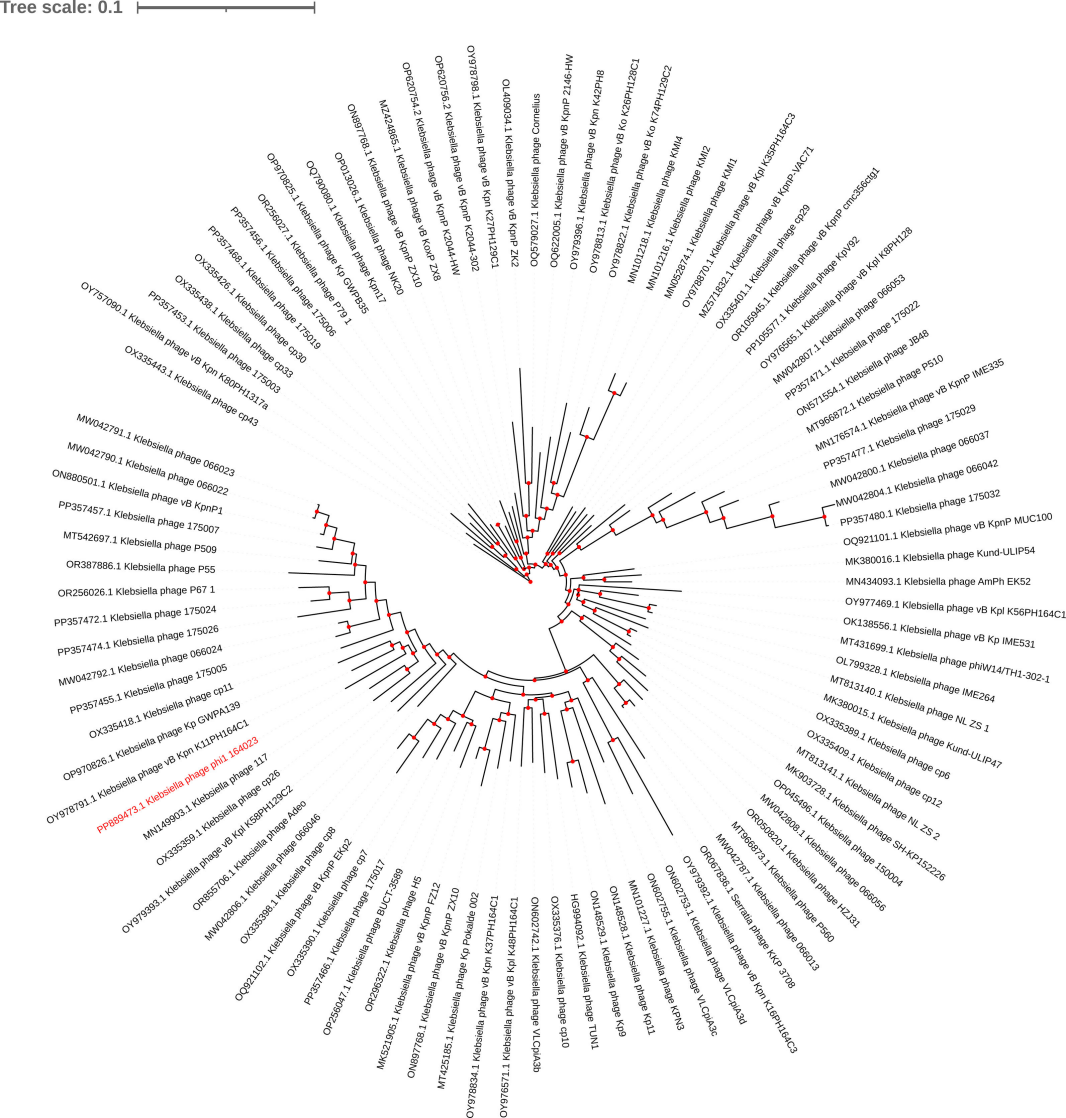
Single-gene phylogeny tree of Przondovirus phages. The maximum likelihood tree was constructed from an alignment of RNA polymerase using IQ-TREE v2.3.6 under the MFP+FU model with 1,000 rapid bootstraps and SH-Alrt tests and was annotated using iTOL v7.

Genome sequencing of phi1_164023 generated a total of 16,840,826 paired-end 150-bp reads, amounting to 2.53 Gb (accession no. SRR36463674. The genome of phi1_164023 is 39,059 bp in length, with a GC content of 53.11%. It was assembled into a single, circular contig using Unicycler, indicating a complete genome, and contains 55 predicted coding sequences but no tRNAs. phi1_164023 carries no antimicrobial resistance genes nor virulence factors and is predicted to have a virulent lifestyle, evidenced by the high (83.6%) BACPHLIP v0.9.3_alpha (10) score. BLAST analysis revealed that phi1_164023 exhibits the highest DNA similarity (91.67%, identity × coverage) with *Klebsiella* phage vB_KpnP_K2044-HW (accession no. OP620754.2). phi1_164023 belongs to the genus *Przondovirus*, subfamily *Studiervirinae*, family *Autotranscriptaviridae*. The maximum likelihood tree was inferred by IQ-TREE v2.3.6 (11) based on RNA polymerase sequences of 97 *Klebsiella* phages belonging to the *Przondovirus* genus in ICTV (https://ictv.global/msl). In this phylogenetic tree, phi1_164023 is indeed clustered with other *Przondovirus* phages (Fig. 1).

## Data Availability

The complete genome sequence of phage phi1_164023 has been deposited in GenBank under accession no. PP889473 and Sequence Read Archive (SRA) accession no. SRR36463674. The version described in this paper is the first version.
